# Efficacy of axillary exclusion on seroma formation after modified radical mastectomy

**DOI:** 10.1186/s12957-016-0801-0

**Published:** 2016-02-20

**Authors:** Mohammed Faisal, Sameh T Abu-Elela, Waleed Mostafa, Osama Antar

**Affiliations:** Department of Surgery-Faculty of Medicine, Suez Canal University, Ismailia, Egypt

**Keywords:** Breast cancer, Seroma, Mastectomy, Axillary exclusion

## Abstract

**Background:**

Breast cancer represented 35.1 % of total female cancer cases in Egypt. Seroma is one of the most serious and common complications of mastectomy and axillary dissection with incidence between 15 and 81 %. Seroma formation delays wound healing and increases susceptibility to infection, skin flap necrosis, and persistent pain as well as prolonging convalescence. Therefore, several techniques have been investigated to minimize seroma formation with no consistent success. Axillary exclusion is a technique aimed to obliterate dead space after axillary clearance and minimize collection.

**Methods:**

Sixty-four patients were prepared for modified radical mastectomy. Of those, the study group contains 32 patients and the control group contains 32 patients. Study group had axillary exclusion while the other had the conventional procedure; total drain outputs were recorded daily for all patients prior to drain removal. The drains were removed when the daily drainage was less than 30 ml.

**Results:**

This study contains 64 patients, the study group contains 32 patients, and the control group contains 32 patients. Age, BMI (mean control = 31.7 and study = 30.2), and tumor size were of no significant differences to be more concise on the effect of axillary exclusion. The mean of day of drain removal in the control group was 17.8 day (15–19) with a mean of total drain output of 4525.6 ml (4430–3660 ml) while the mean in the study group of day of drain removal was 11.3 (10–13) with a mean of total drain output of 1476.2 ml (620–2200 ml), *p* < 0.00.

**Conclusions:**

Axillary exclusion technique is a valuable procedure that significantly decreases seroma postmastectomy and axillary dissection.

## Background

Breast cancer is the most common malignancy among women in most developed and developing regions of the world with nearly a million new cases each year [1]. In a recent epidemiological study in the Egyptian cancer institute, breast cancer represented 35.1 % of the total female cancer cases in Egypt [2]. Modified radical mastectomy is the most common form of breast cancer treatment [3]. Patients at a higher risk for postoperative complications are patients with diabetes, smokers, patients with a history of prior chest wall radiation, and other patients with diffuse small vessel disease. After an axillary dissection, along with the normal local healing issues, the alteration of the regional lymphatic system puts patients at an increased risk of complications [4]. The incidence of seroma is correlated with certain factors. Obesity, patient’s age, hypertension, breast volume, presence of malignant nodes in the axillary region, number of metastatic nodes, number of dissected nodes, early shoulder exercise, and the use of some drugs, i.e., tamoxifen and heparin, affect the pathophysiology. While the use of electrocautery decreases bleeding, it increases total drain output, causing a higher rate of seroma formation [5, 6]. Theories of etiology are important in determining the most likely surgical technique for prevention. Various techniques have been studied in an attempt to minimize postmastectomy drainage volumes and the incidence of seroma. None however have been found to be consistently successful, and consequently, none are used in the common practice. If it is believed that the lymphatics disruption in the axillary fossa are main aetiology, it follows that obliterating this space will minimize fluid collection [7, 8].

## Methods

This study is a randomized controlled trial that took place in Surgery Department, Suez Canal University Hospital, from November 2013 to August 2014. This research has been reviewed by our research ethics committee in the Faculty of Medicine-Suez Canal University at its meeting on 23/4/2014 with reference number (#2115).

Target population was 64 patients among those who referred to outpatient clinics. The sample size was calculated as 32 patients for each group, using MedClac Version 11.4 software. This number of patients was large enough to detect a difference in the mean “number of days before drain removal” of 20 days (±23.5 days pooled standard deviation), at 5 % alpha error, 10 % beta error, and 10 % dropout rate.

**Fig. 1:**
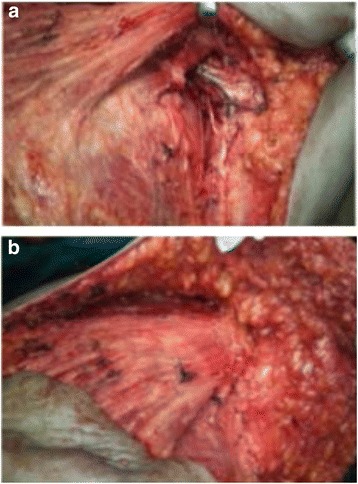
Study intra operative images show (**a**) axillary fossa after mastectomy and axillary clearance and (**b**) axillary exclusion: the technique was performed by a single surgeon and involved skin flap dissection and the excision of the breast with pectoral fascia, and the dissection of axillary lymph nodes were performed with a diathermy (**a**). Control of the small bleeding vessels was sustained with coagulate mood of diathermy. Suturing the superior mastectomy skin flap down to the free edge of pectoralis major and the lateral chest wall was done using a continuous 2/0 vicryl stitch, and then, four to six interrupted sutures were placed between pectoralis major and minor to reliably exclude the axillary fossa from the remainder of the mastectomy cavity (**b**)

Those 64 patients were recruited to our study according to the inclusion and exclusion criteria. Inclusion criteria were any patients presented to the surgery outpatient clinic with breast cancer and planned for modified radical mastectomy, and exclusion criteria were (1) patients arranged for conservative breast surgery and sentinel lymph node, (2) patients with advanced breast cancer and arranged for palliative mastectomy, and (3) patients arranged for breast reconstruction at the same session. Then, they were randomly allocated to either the control or study groups. Random sequence was generated by Microsoft Excel program using random functions. The surgeon was given randomly generated treatment allocations within sealed opaque envelopes. Once a patient has consented to enter the trial, this envelope was opened and the patient then underwent the allocated surgery Thirty-two patients were equally and randomly assigned as the study group, and the control group contains thirty-two patients. The technique was performed by a single surgeon and involved skin flap dissection and excision of the breast with pectoral fascia, and the dissection of axillary lymph nodes were performed with a diathermy (Fig. 1a). Control of the small bleeding vessels was sustained with coagulate mood of diathermy. Suturing the superior mastectomy skin flap down to the free edge of pectoralis major and the lateral chest wall was done using a continuous 2/0 vicryl stitch; then, four to six interrupted sutures were placed between pectoralis major and minor to reliably exclude the axillary fossa from the remainder of the mastectomy cavity (Fig. 1b). 14F suction drains were placed at surgery in all patients with the tip placed within the mastectomy cavity outside the obliterated axilla; then, pressure dressing was applied. Total drain outputs were recorded daily for all patients prior to drain removal. The drains were removed when the daily drainage was less than 30 ml.

## Results

**Fig. 2:**
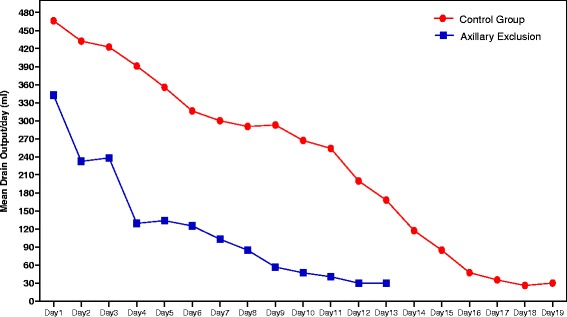
Distribution of patients according to daily drain output (ml). Significant reduction of the daily amount of the seroma in the study group who underwent axillary exclusion, *p* < 0.05

**Fig. 3:**
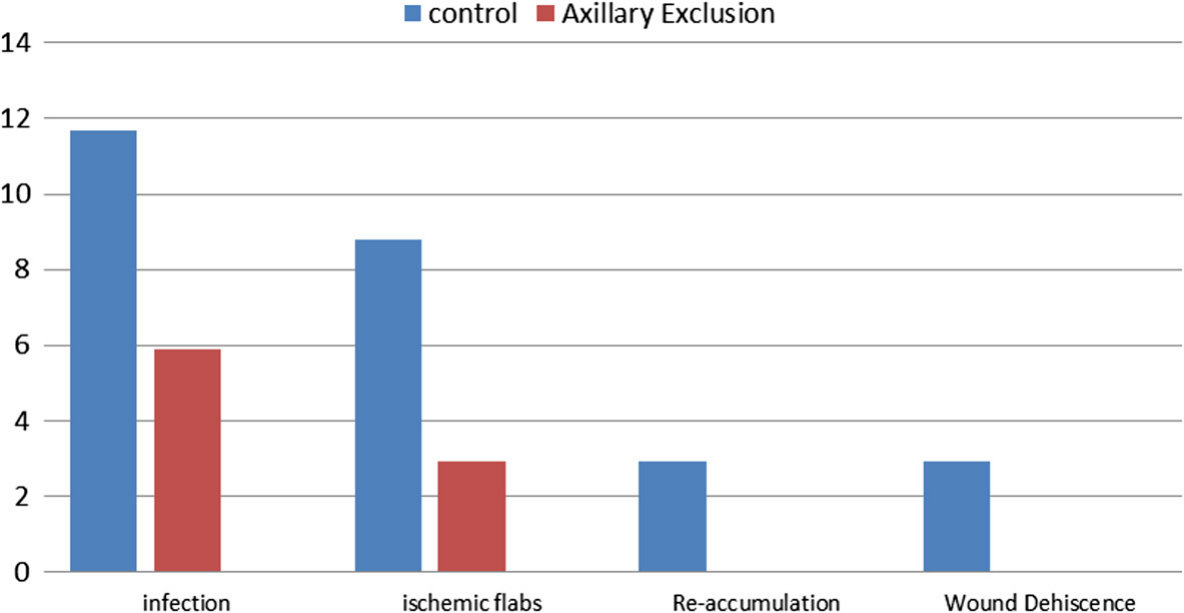
Distribution of patients according to postoperative complications. For the distribution of the participants according to the list of postoperative complications in both groups, in the study group, there was 91.2 % of patients with no postoperative complications, 5.9 % developed wound infection, 2.9 % developed ischemic flaps, and there was no one developed reaccumulation or wound dehiscence while the control group showed 73.8 % of patients with no postoperative complications, 11.7 % developed infection, 8.8 % developed ischemic flaps, 2.9 % reaccumulation, and 2.9 % developed wound dehiscence

Both study groups contains and control group comprised 32 patients. The mean age in the study group was 48.9 ± 4.1 years versus 47.5 ± 2.3 years in the control group with no significant difference. The mean BMI of the study group, 30.2 ± 2 %, did not show any significant statistical difference from the control group, 31.7 ± 1 %. Similarly, the mean tumor size in the study group, 3 × 2.2 cm, did not differ significantly than the control group 2.9 × 2.8 cm. The total amount of the drain output was compared in both groups. The mean total amount in the study group was 1476.2 ± 518 ml while the mean in the control group was 4525.6 ± 97.6 ml. The results show a significant reduction in the total amount of the drain output *p* < 0.001 (Table 1). There was a significant reduction in the daily amount of the seroma in the study group who underwent axillary exclusion *p* < 0.05 (Fig. 2). There was a significant difference in the results between the study and the control group regarding the day of the drain removal as the mean of the days before drain removal in study group was 11.3 ± 1.3 day while in the control group was 17.8 ± 1 day *p* < 0.001 (Table 2). For the distribution of the participants according to the list of postoperative complications in both groups, in the study group, there was 91.2 % of patients with no postoperative complications, 5.9 % developed wound infection, 2.9 % developed ischemic flaps, and there was no one developed reaccumulation or wound dehiscence while the control group showed 73.8 % of patients with no postoperative complications, 11.7 % developed infection, 8.8 % developed ischemic flaps, 2.9 % reaccumulation, and 2.9 % developed wound dehiscence (Fig. 3).

**Table 1:** Distribution of patients according to total drain output

	Study groups						*p* value
	Control (*n* = 32)			Axillary exclusion (*n* = 32)			
	Mean	±SD	Range	Mean	±SD	Range	
Total drain output (ml)	4525.6	97.6	4430–3660	1476.2	518	620–2200	<0.001*

*Statistically significant at *p* < 0.05

**Table 2:** Distribution of patients according to day of drain removal

	Study groups						*p* value
	Control (*n* = 32)			Axillary exclusion (*n* = 32)			
	Mean	±SD	Range	Mean	±SD	Range	
Days before drain removal	17.8	1.0	15–19	11.3	1.3	10–13	<0.001*

*Statistically significant at *p* < 0.05

## Discussion

Axillary dissection remains an integral part of breast cancer treatment for prognostic and curative purposes [9]. It is possible to avoid axillary dissection in selected patients (T1N0) using the sentinel lymph node technique. However, in the majority of cases, axillary lymphnectomy is not avoidable and still has complications, in particular seroma formation (15–81 %), which can delay the patient’s discharge, healing, and supplementary radiotherapy and chemotherapy treatments [10, 11]. Various studies have attempted to reduce seroma formation in order to improve outcome and reduce morbidity. Techniques that have been advocated over the years include shoulder immobilization, prolonged suction drainage perioperative tranexamic acid, choice of surgical instrument, and obliteration of dead space [12]. Electrocautery has been described as possibly increasing the frequency of seroma. Contrary to the popular belief, a study has shown that the length of time drains that are left in place does not affect seroma rate. Few results have shown consistent benefit time of initiation of arm movement has also been studied on the basis that chest wall motion and shoulder use create shearing forces that delay flap adherence and that postoperative arm use acts as a pump forcing lymph into the empty axillary fossa [13]. However, studies have shown no significant difference when delaying rehabilitation, and in fact, the consequences of shoulder stiffness can be far greater than that of simple seroma [14, 15]. We believe that postoperative fluid collections following mastectomy and axillary clearance arise from disrupted axillary lymphatics to a greater extent than serous fluid formation from mastectomy flaps. We have shown that reliably excluding the axillary fossa from the remainder of the mastectomy wound can considerably reduce postoperative drainage volume in this small group of patients. More importantly, this technique significantly reduces clinically apparent seromas after drain removal, thereby reducing the consequences of patient anxiety, discomfort, and added morbidity [16]. Previous studies of seroma formation after breast surgery have often been small in scale and poor in quality, and none have clearly demonstrated a difference in overall patient-rated quality of life or cost-effectiveness as a result of mechanical closure of the dead space (Classe JM et al. 2006). However, the findings suggest that routine use of a pressure garment or compression dressing is not warranted. In contrast, closure of the dead space by flap fixation with sutures will reduce seroma formation and the number of aspirations, thus simplifying postoperative management and facilitating early discharge. This technique may preclude the use of drains in breast surgery, especially in BCS [17].

Yiping Gong, MB, [18], studied 200 breast cancer patients randomly divided into 2 groups: group 1 was operated by using ligation all of the tissue connecting axillary vein bundles to the specimen, suturing the anterior edge of the latissimus dorsi to the chest wall, and fix the skin flap to the underlying muscle by subcutaneous sutures while group 2 was operated on using the conventional technique. The drainage volume, in the initial 3 days, for patients in group 1 was significantly less than that for patients in group 2 (*P* < 0.01). The duration of drainage in group 1 was shorter than that in group 2 (*P* < 0.01). The incidence of seroma formation in the study group (2 %) was significantly less than that in group 2 (14 %) (*P* < 0.01).

## Conclusions

Axillary exclusion is a simple technique that reduces significantly the total amount of seroma formation postoperatively, the psychological burden of long time drain, postoperative visits for drain follow-up, and complications related to seroma accumulation after breast cancer surgery. However, we recommend that this technique should be tried on a much wider scale to prove its validity in decreasing the incidence of seroma formation and its subsequent complications, so that it can be introduced as a step in the mastectomy operations.
